# Diagnostic accuracy of lung ultrasound in children with respiratory pathology admitted in a pediatric intensive care unit of a low-resource setting: a single-center experience

**DOI:** 10.62675/2965-2774.20260146

**Published:** 2026-04-16

**Authors:** Qalab Abbas, Farah Khalid, Haania Rizwan, Uswah Siddiqi, Areesh Mevawalla, Arsheen Zeeshan, Fyezah Jehan

**Affiliations:** 1 Aga Khan University Department of Pediatrics and Child Health Karachi Pakistan Department of Pediatrics and Child Health, Aga Khan University - Karachi, Pakistan.; 2 Aga Khan University Medical College Karachi Pakistan Medical College, Aga Khan University - Karachi, Pakistan.

**Keywords:** Ultrasonography, Lung diseases, Respiratory diseases, Chest x-ray, Child, Pediatric intensive care units

## Abstract

**Objective:**

To determine the sensitivity and specificity of lung ultrasound in diagnosing respiratory pathologies in children on respiratory support in the pediatric intensive care unit, compared to chest X-ray and clinical diagnosis.

**Methods:**

A cross-sectional study was conducted on children aged 1 month to 18 years admitted to the pediatric intensive care unit and requiring respiratory support from June 2018 to February 2019. Lung ultrasound was performed within 24 hours of chest X-ray by a trained sonographer using standardized protocols. Lung ultrasound and chest X-ray were interpreted independently by blinded physicians. A Receiver Operating Characteristic curve was generated to assess lung ultrasound diagnostic performance using chest X-ray as the gold standard.

**Results:**

A total of 220 lung ultrasounds were performed on 117 patients, with 195 (88.6%) examinations completed. Lung ultrasound and chest X-ray were reported normal in 24 (10.9%) and 21 (9.5%) studies, respectively, with no pneumothorax detected. Overall, lung ultrasound had a sensitivity of 89.95% and specificity of 19.05% compared to chest X-ray. Sensitivity and specificity for pneumonia and pediatric acute respiratory distress syndrome were 62.8% and 44.8%, and 50% and 96%, respectively. Using clinical diagnosis as reference, sensitivity and specificity for pneumonia and pediatric acute respiratory distress syndrome were 59.8% and 54.3%, and 72.7% and 95.7%, respectively. Agreement between chest X-ray and lung ultrasound was poor (k = 0.085), though concordance among lung ultrasound providers was high (k = 0.869). Agreement for lung ultrasound and pediatric acute respiratory distress syndrome was highest (k = 0.632) compared with clinical diagnosis. Receiver Operating Curve analysis showed that lung ultrasound showed poor diagnostic accuracy compared to chest X-ray (AUC 0.54).

**Conclusion:**

Lung ultrasound is feasible in low-resource pediatric intensive care unit settings but shows limited diagnostic accuracy compared to chest X-ray.

## INTRODUCTION

Respiratory deterioration is a leading cause of pediatric intensive care unit (ICU) admissions, accounting for approximately 20 - 27% of all pediatric ICU admissions.^(1)^ Portable chest X-rays (CXR) are commonly used due to their accessibility and speed; however, they have limitations such as radiation exposure, low sensitivity for detecting small pleural effusions and pneumothorax, and delays in decision making.^(2,3)^ Although computed tomography (CT) of the chest serves as the gold standard for diagnosing lung pathologies, its practical application in the pediatric ICU is limited due to transport-related risks, radiation exposure concerns, and substantial costs.

Lung ultrasound (LUS) has emerged as a promising alternative bedside imaging tool, with demonstrated higher sensitivity in diagnosing pneumonia and other pulmonary pathologies in adults and children,^(4–8)^ while also assisting with various procedures. It has also been shown to be more sensitive than supine CXR as a diagnostic tool for pneumothorax, small effusions,^(9)^ consolidation, and ventilator-associated pneumonia with a positive predictive value of 86%.^(10)^ However, data on the efficacy of LUS in diagnosing pulmonary pathologies in a diverse population of critically ill children admitted to a pediatric ICU are limited. A study by Tripathi et al. showed a sensitivity of 82% and a specificity of 25% in detecting underlying pulmonary pathologies in children admitted to the pediatric ICU.^(4)^ Another study by DeSanti et al. showed that LUS correctly identified the etiology of acute respiratory failure in 56% of patients in the pediatric ICU.^(11)^

However, its diagnostic performance in the diverse pediatric ICU population remains unexplored, and its broader adoption is hindered by variability in image acquisition and interpretation. We hypothesize that LUS is both as feasible and at least as accurate as routine methods for diagnosing respiratory pathologies in critically ill children. The primary objective of this study is to determine the sensitivity and specificity of LUS in diagnosing respiratory pathologies in children receiving respiratory support, compared with CXR and clinical diagnosis. Secondary objectives are to determine the feasibility of LUS and explore its potential as a primary diagnostic tool in a low-resource pediatric ICU setting.

## METHODS

### Study settings

A cross-sectional analysis was conducted in a private tertiary pediatric ICU in Pakistan. Lung ultrasound examinations were performed only on hemodynamically stable patients with physician approval and discontinued if patients became unstable. The clinical team remained blinded to LUS results, which did not influence treatment decisions. The protocol adhered to the Declaration of Helsinki and local regulations, with LUS methodology piloted before study initiation. The Aga Khan University Ethics Committee exempted the study from written consent requirements (ERC# 2019-1265-3129).

### Patient enrolment

Lung ultrasound was introduced in the pediatric ICU in early 2018. Initially, it was implemented without formalized reporting or associated costs and was used alongside CXR, which were performed as needed as part of standard routine care. All patients aged 1 month to 18 years admitted to the pediatric ICU from June 2018 to February 2019 with signs and symptoms of respiratory distress or requiring respiratory support (noninvasive or invasive), who had a CXR performed were included in the study. Respiratory distress and respiratory failure were defined as per Pediatric Advanced Life Support guidelines.^(12)^ Patients who did not have a CXR done during the pediatric ICU stay and those with chronic lung disease were excluded from this study. Lung ultrasound was performed within 24 hours of the CXR. If multiple CXR were performed within that 24-hour window, the most recent CXR was used for comparison with the LUS. Patient data was deidentified and stored in a password-protected computer.

### Lung ultrasound procedure

All LUS examinations were performed using the Philips^®^ Sparq ultrasound machine (Philips^®^, Koninklijke Philips N.V., Amsterdam, the Netherlands) using a 5 - 12MHz linear array transducer by a single trained sonographer. The sonographer received additional 1-day standardized training in performing LUS in children, in accordance with the American College of Radiology and Society of Pediatric Radiology (ACR-SPR) guidelines. This was followed by 3 days of supervised scanning and interpretation under the guidance of a radiologist at Aga Khan University Hospital. The training took place prior to recruitment for the study and followed a methodology similar to that of Bueno-Campaña et al., to ensure consistency in examinations across all participants.^(13)^ The participating sonographer had prior experience performing and interpreting LUS from another study before joining this one.^(14)^

Lung ultrasound was performed in the most comfortable position for awake patients, i.e., in the mother's lap or lying on the bed. Sedated patients had LUS performed in the supine position, followed by lateral and posterior images obtained by changing their position to the lateral side. In cases in which an image could not be acquired or patient repositioning was not possible due to chest drain placement or hemodynamic or respiratory instability, the examination was labeled as limited, and only approachable areas of the lungs were imaged. A 3 - 5 second clip was stored, and gain and depth were adjusted as per patient size. The ultrasound probe was placed perpendicular to the ribs to obtain longitudinal images. Both video and still images were used to detect radiological findings, while measurements were taken from still images. In cases of abnormal findings, the ultrasound probe was also placed parallel to the ribs to obtain transverse images.

**Figure 1:**
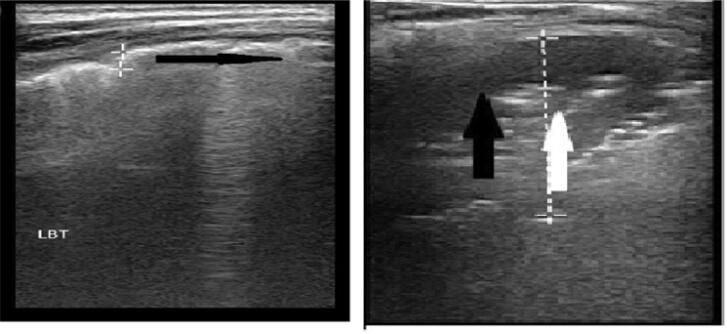
Normal and abnormal lung ultrasound findings.

Lung ultrasound imaging was performed in six zones on each hemithorax, following a previously published protocol adapted for our study.^(4)^ Each hemithorax was divided into anterior, lateral, and posterior zones, with each zone further subdivided into superior and inferior regions. The nipple served as the boundary between the superior and inferior regions, while the anterior and posterior axillary lines defined the borders between the anterior, lateral, and posterior lung zones. Ultrasound findings were categorized according to established definitions. The pleural line was defined as a hyperechoic line below the rib line. A-lines appeared as hyperechoic horizontal lines parallel to the pleural line, created by reflection of ultrasound waves between the pleura and the transducer. B-lines were identified as discrete, laser-like vertical hyperechoic lines originating from the pleural line, extending to the bottom of the screen, and moving synchronously with lung sliding. When multiple B-lines became non-discrete and originated from pleural line artifacts, they were classified as confluent B-lines. Significant consolidation was diagnosed when subpleural areas showed loss of A-lines with hypoechoic, tissue-like echo texture containing air bronchograms (hyperechoic punctiform images). Similar parenchymal tissue-like appearance without air bronchograms was classified as atelectasis.^(6)^ Imaging began with an assessment of lung sliding bilaterally in both B-mode and M-mode across all zones to rule out pneumothorax. A comprehensive evaluation for pleural line irregularities, including the presence of Z-lines, A-lines, B-lines, or a C-pattern, followed (Figure 1). All examinations were extended on the lateral and posterior zones to see the lung base and visualize the diaphragm to look for pleural effusion (Tables 1S and 2S and Figure 1S - Supplementary Material).

Image 1 shows that the that the A-line (black arrow) is typically horizontal, with equidistant artifacts arising from the reflection of ultrasound waves between the transducer and the pleura in the presence of air. In this image, faint A-lines appear. These horizontal repetitions are spaced at a regular interval below the pleural line, which is the bright horizontal line near the top of the image. A-lines are typically indicative of normal, air-filled lung tissue.

Image 2 shows the absence of A-lines; as in the previous image, A-lines, which are indicative of a normal air-filled lung, are not prominently seen. Multiple vertical artifacts extend from the pleural line to the bottom of the image. These are B-lines, indicative of an interstitial process. The presence of multiple B-lines in this image may suggest conditions such as pulmonary edema, interstitial lung disease, or other lung pathologies. There is a region below the pleural line that appears to be hypoechoic (darker). This might suggest lung consolidation or atelectasis, indicating an area where the lung tissue has become denser or more solid.

### Lung ultrasound interpretation

Interpretation of LUS was done by a trained sonologist and reported by a sonologist and pediatric ICU physician. The reporting physician had completed the Pediatric Ultrasound Course by the Society of Critical Care Medicine and had been credentialed after performing 20 supervised LUS examinations prior to this study. Both interpreters met the competency criteria described by the American College of Chest Physicians (ACCP) and the ACR-SPR consensus statement on Competence in Critical Care Sonography for Methodological Standardization.^(15)^ To minimize bias, patients were enrolled only when the study physician was not on call in the pediatric ICU. As a result, the physician had no direct or indirect involvement in patient care and remained blinded to CXR findings and other clinical details. Lung ultrasound findings were recorded on a structured pro forma for LUS interpretation. To further standardize reporting of pathology, the sonologist and pediatric ICU attending categorized the final diagnostic findings as one or more of the following eight categories: bronchiolitis, pneumonia, atelectasis, pulmonary edema, pleural effusion, normal, pediatric acute respiratory distress syndrome (PARDS) and interstitial lung pathology, or other according to BLUE protocol criteria and Dietrich et al.^(16,17)^ In cases of disagreement over the final pathology, images were reviewed by another ACR-SPR accredited radiologist who served as a tiebreaker. Chest X-ray images were interpreted by a pediatric radiologist following Schneider's guidelines for CXR interpretation (Table 3S - Supplementary Material).^(18)^ The radiologist was blinded to LUS imaging and findings to prevent bias and facilitate accurate comparison between the two modalities.

A comparative analysis was conducted using follow-up LUS examinations to evaluate disease progression in patients remaining in the pediatric ICU, and the findings were subsequently compared with the corresponding CXR. The study examined three clinical conditions: pneumonia, bronchiolitis, and PARDS. Attending physicians were later consulted to provide their clinical assessments and provisional diagnoses, which were evaluated for consistency with the LUS results. All clinical observations and diagnostic outcomes were recorded.

### Statistical analysis

Statistical analysis was performed using Stata^TM^ version 15.0. Data were presented as frequencies and percentages for qualitative variables and median and interquartile range (IQR) for quantitative variables. Diagnostic accuracy was computed using sensitivity, specificity, positive predictive value, and negative predictive value with 95% confidence interval (95%CI). The area under the curve (AUC) was calculated from the Receiver Operating Characteristic (ROC) curve to assess the predictive ability of LUS using CXR as the gold standard. Kappa statistics were used to assess the agreement between two ultrasound and CXR providers, as well as the agreement between the two diagnostic methods and between LUS diagnosis and clinical diagnosis.

In addition to frequentist methods, a Bayesian analysis was performed using non-informative Beta (1,1) priors for the parameters of interest. Posterior estimates of sensitivity, specificity, likelihood ratios, and predictive values were derived from the observed 2×2 table, with 95% credible intervals obtained from posterior distributions. This approach is well-suited for small or imbalanced samples.

## RESULTS

A total of 220 LUS were performed on 117 patients during the study period, with 195 (88.6%) LUS examinations completed successfully. Basic demographic and clinical characteristics of the study population are shown in table 1. Nine patients were hemodynamically unstable, four had more than one chest drain placed, five patients were on high ventilatory parameters, and seven patients were awake and irritable, so their examination was incomplete. Lung ultrasound was performed once in 57 patients (48.7%), twice in 36 patients (30.7%), and three or more times in 24 patients (20.5%). The median age of patients was 11 months (IQR 4 - 36). Lung ultrasound was performed while patients were on invasive respiratory support in 115 (52.7%) cases.

**Table 1:** Baseline characteristics of study population

Variables		
Total patients		117
Total ultrasound		220
Ultrasound completion		195 (88.6)
Clinical diagnosis (n = 117)		
	Respiratory disease	79 (67.5)
	Cardiovascular system	27 (23)
	Central nervous system	3 (2.3)
	Infection/sepsis	4 (3.6)
	Miscellaneous	4 (3.6)
Age in months		11 (4 - 36)
Age (n = 117)		
	< 2 months	12 (10.25)
	2 - 12 months	48 (41.02)
	1 - 5 years	34 (29.05)
	> 5 years	23 (19.6)
Gender (n = 117)		
	Male	73 (62.3)
	Female	44 (37.7)
Invasive respiratory support		116 (52.7)
Lung ultrasound results		
	Normal	24 (10.9)
	Pediatric acute respiratory distress syndrome	18 (8.2)
	Pneumonia	128 (58.2)
	Bronchiolitis	10 (4.5)
	Pulmonary edema	5 (2.3)
	Atelectasis	28 (12.7)
	Pleural effusion	2 (0.9)
	Others	5 (2.3)
Chest X-ray results		
	Normal	21 (9.5)
	Pediatric acute respiratory distress syndrome	20 (9.1)
	Pneumonia	86 (39.1)
	Bronchiolitis	1 (0.5)
	Pulmonary edema	17 (7.7)
	Atelectasis	54 (24.5)
	Pleural effusion	12 (5.5)
	Others	9 (4.1)

Results expressed as n, n (%) or median (interquartile range).

Common LUS-based diagnoses were pneumonia (128; 58.2%), atelectasis (28; 12.7%), and PARDS (18; 8.2%), while the most common diagnoses based on CXR findings were pneumonia (86; 39.1%), atelectasis (54; 24.5%), and PARDS (20; 9.1%). There was no case of pneumothorax as a diagnosis in our cohort.

**Figure 2:**
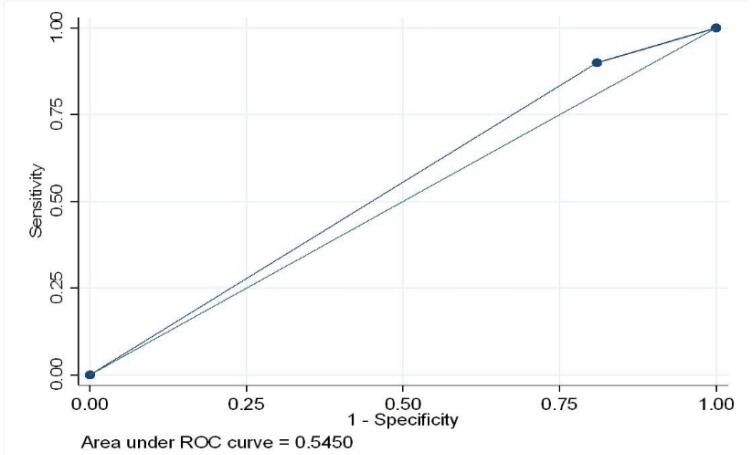
Area under the curve for overall ultrasound performance (ultrasound *versus* X-ray).

Using CXR as a reference standard, the overall sensitivity and specificity of LUS were 89.95% and 19.05%, respectively (AUC = 0.54) (Figure 2 and Table 2). When stratified by diagnoses, the observed sensitivity and specificity were 50% and 96.0% for PARDS and 62.80% and 44.8% for pneumonia (Table 3).

ROC - Receiver Operating Characteristic.

**Table 2:** Sensitivity and specificity analysis for lung ultrasound compared with chest X-ray

Categories		Sensitivity %	Specificity %	PPV %	NPV %
Overall		89.95	19.05	91.30	16.7
Age					
	< 2 months	85.71	0.00	94.7	0.0
	2 - 12 months	92.94	0.00	92.90	0.00
	1 - 5 years	86.44	20.00	92.7	11.10
	> 5 years	91.18	33.33	83.80	50.00
Diagnosis					
	Pediatric acute respiratory distress syndrome	50.00	96.00	55.60	95.00
	Pneumonia	62.80	44.80	42.20	65.20
	Bronchiolitis	0.00	95.40	0.00	99.50
	Pulmonary edema	0.00	97.50	0.00	92.10
	Atelectasis	11.10	86.70	21.40	75.00
	Pleural effusion	0.00	99.00	0.00	94.50

PPV - positive predictive value; NPV - negative predictive value.

**Table 3:** Sensitivity and specificity analysis for lung ultrasound, taking clinical diagnosis as a gold standard

Categories		Sensitivity %	Specificity %	PPV %	NPV %
Diagnosis					
	Pediatric acute respiratory distress syndrome	72.7	95.70	61.50	97.40
	Pneumonia	59.80	54.30	77.50	33.90
	Bronchiolitis	11.10	94.50	25.00	86.60

PPV - positive predictive value; NPV - negative predictive value.

In patients aged 2 - 12 months, LUS had the highest sensitivity (92.94%) and a positive predictive value of 92.9%. In patients older than 5 years, LUS sensitivity was 91.18%, the second highest among age groups. Overall, sensitivity was highest for patients aged 2 - 12 months, whereas specificity was highest for patients aged 5+ (33.33%). The sensitivity analysis for LUS was also repeated using clinical diagnoses of PARDS, pneumonia, and bronchiolitis as gold standards. Pediatric acute respiratory distress syndrome showed the highest sensitivity and specificity, at 72.7% and 95.7%, respectively (Table 3).

The agreement between CXR providers using kappa statistics was excellent (k = 0.952; 95%CI 0.940 - 0.970), whereas the agreement between LUS providers was also excellent (k = 0.869; 95%CI 0.850 - 0.905). However, the overall agreement between CXR and LUS was inferior (k = 0.085; 95%CI: -0.077 - 0.246). The highest agreement was noted for the age group of greater than 5 years (κ 0.279; 95%CI 0.087 - 0.471), and the lowest agreement was noted for the age group 2 - 12 months (κ -0.071; 95%CI -0.163 - 0.022). The agreement was lowest when the diagnosis was bronchiolitis, -0.008 (95%CI -0.025 - 0.008), and highest at 0.482 (0.277 - 0.687) for the diagnosis of PARDS (Table 4).

**Table 4:** Agreement statistics

Variable		Kappa	95%CI
Overall		0.085	(-0.077 - 0.246)
Chest X-ray and lung ultrasound			
	Pediatric acute respiratory distress syndrome	0.482	(0.277 - 0.687)
	Pneumonia	0.07	(-0.052 - 0.192)
	Bronchiolitis	-0.008	(-0.025 - 0.008)
	Pulmonary edema	-0.036	(-0.069 - -0.004)
	Atelectasis	-0.026	(-0.143 - 0.092)
	Pleural effusion	-0.016	(-0.038 - 0.006)
Clinical diagnosis with lung ultrasound			
	Pediatric acute respiratory distress syndrome	0.632	(0.398 - 0.867)
	Pneumonia	0.119	(-0.045 - 0.282)
	Bronchiolitis	0.073	(-0.122 - 0.268)
Clinical diagnosis with chest X-Ray			
	Pediatric acute respiratory distress syndrome	0.794	(0.620 - 0.967)
	Pneumonia	0.294	(0.146 - 0.442)
	Bronchiolitis	0.000	(0.00 - 1.000)
Age			
	< 2 months	-0.073	(-0.164 - 0.017)
	2 - 12 months	-0.071	(-0.163 - 0.022)
	1 - 5 years	0.047	(-0.103 - 0.197)
> 5 years		0.279	(0.087 - 0.471)
	Between chest X-ray provider 1 and provider 2	0.952	(0.940 - 0.970)
	Between lung ultrasound provider 1 and provider 2	0.869	(0.850 - 0.905)

95%CI - 95% confidence interval.

Agreement between clinical diagnoses of PARDS, pneumonia, and bronchiolitis was also established, with LUS and CXR diagnoses assessed separately; PARDS showed the highest agreement with both. The kappa statistics for LUS diagnosis of PARDS were k = 0.632 (95%CI 0.398 - 0.867), whereas the kappa statistics for CXR diagnosis of PARDS were k = 0.794 (95%CI 0.620 - 0.967). Overall, the ROC analysis demonstrated suboptimal diagnostic discrimination of LUS using CXR as the gold standard, with an AUC of 0.54 (Figure 2).

Bayesian analysis results also revealed low specificity (21.80%; 7.9% - 40.5%), a ROC area of 0.57 (0.46 - 0.67), and negative predictive value (NPV) of 44.40% (13.7% - 78.8%) (Table 4S - Supplementary Material).

## DISCUSSION

This study evaluated the diagnostic accuracy of point-of-care LUS compared with CXR and clinical diagnosis for detecting pulmonary pathology in critically ill children requiring respiratory support in the pediatric ICU. Our findings showed that LUS demonstrated suboptimal overall diagnostic accuracy and agreement with CXR, particularly pneumonia, while it may have potential value for specific conditions like PARDS. The overall completion rate was excellent, and performance improved with age. Its performance was also better when compared with clinical diagnosis and suboptimal when compared to CXR.

The overall agreement between CXR and LUS findings was very low or almost non-existent (kappa = 0.085), lower than previously reported in adults and pediatric populations.^(1,2)^ The highest level of agreement was observed for PARDS (k = 0.48). This was still lower than the good agreement observed between CXR and clinical diagnosis of PARDS (k = 0.794), which is in line with current PALICC-2 guidelines, which recommend using radiographic findings in the PARDS definition.^(19)^ This was also confirmed by Bayesian analysis, indicating this is not an artifact of the statistical method used. These discrepancies further reinforce the importance of LUS interpretation within the clinical context, rather than relying on it as a standalone diagnostic tool. Compared to clinical diagnosis, LUS showed moderate diagnostic performance for PARDS (sensitivity and specificity of 72.7% and 95.7%, respectively) but suboptimal sensitivity and specificity (59.8% and 54.3%, respectively) for pneumonia. Notably, LUS detected pneumonia in 128 examinations, compared with 86 by CXR, likely due to LUS's ability to detect B-lines and subpleural blebs that are undetectable on radiographs, though subjective interpretation remains a concern. This is in contrast with previous studies; for example, DeSanti et al. showed a sensitivity of 76% for LUS in diagnosing pneumonia when compared to clinical assessment, whereas our study showed a sensitivity of 59.8%.^(11)^ These contrasting results may be due to differences in patient populations (age, case-mix) or operator experience.

An earlier study in the pediatric ICU by Tripathi et al. reported a 95% completion rate for examinations, compared to 88% in our study.^(4)^ Half of our study population was under 1 year of age, and the majority had underlying respiratory or post-operative cardiovascular conditions, which made it challenging to complete the LUS examination, particularly in the posterior regions that require changes in patient positioning. Despite these challenges, the overall sensitivity of LUS was 89%, which is higher than that shown by Tripathi et al. (82% by operator and 63% by the reader).^(4)^ The highest LUS sensitivity and specificity compared to CXR were found to be for pneumonia (62% and 44%, respectively), followed by PARDS (50% and 96%). These results are in contrast with a meta-analysis on LUS for the diagnosis of pneumonia in children, which showed an overall sensitivity and specificity of 96% and 93%, respectively.^(7)^ Our results showed less sensitivity and significantly lower specificity.

Atelectasis was the second most common positive finding after pneumonia on LUS (12.7%) and CXR (24.5%). Lung ultrasound was very specific for atelectasis, but the sensitivity might be low because of the low number of cases in these categories; therefore, these findings need to be interpreted with caution. Our findings highlight several important considerations for LUS implementation in pediatric critical care. The high specificity for PARDS (96%) suggests that positive LUS findings for this condition are highly reliable, and it can be used to monitor disease progression and treatment response. Conversely, the lower specificity for pneumonia (54.3%) suggests that LUS positive findings should be interpreted cautiously and correlated with clinical presentation and other diagnostic modalities.

Lung ultrasound is a modality with the potential to replace CXR for chest imaging;; however, there are many considerations before standard implementation. Standardized protocols, such as the BLUE protocol for diagnosing acute respiratory failure, are essential for consistent image acquisition and interpretation.^(16)^ While there are training guidelines for physician proficiency in LUS,^(20)^ the number of examinations required to complete the learning curve remains undefined. In pediatric ICU settings, significant delays in obtaining images can occur when portable CXRs and technicians are not readily available; therefore, LUS may be a better alternative.

Our findings reiterate that LUS should be used in combination with clinical history and examination findings to increase its diagnostic accuracy, especially in emergent situations where repeat examination is required. Research has shown that LUS is nearly equivalent to CT in detecting most respiratory pathologies. However, there are limitations to its use in situations like the pediatric ICU.^(21)^ Lung ultrasound is a cost-effective adjunct to diagnosing respiratory pathologies and can potentially be used as an alternative to CXR for pathologies like pneumonia and PARDS. The growing body of evidence and increasing consensus on the sonographic features of respiratory pathologies support the use of LUS as a cost-effective alternative to CXR for conditions such as pneumonia and PARDS, and as a complementary tool for other diagnoses.^(22,23)^ Though LUS is more practical in comparison to portable CXR as a point-of-care tool to assess and manage patients in real-time, future studies should focus on developing a standardized and comprehensive protocol for widespread implementation in critically ill children in the pediatric ICU. Moreover, increasing the implementation of LUS also requires a standardized training program to enhance the standard of care.^(20,24,25)^

### Limitations

There were several important limitations in our study. A key limitation was the use of CXR as the reference standard rather than CT, which is the agreed-upon gold standard for pulmonary imaging. In our resource-limited setting, CT was not feasible for routine use due to financial constraints, leaving CXR as the next best choice despite its known limitations in sensitivity and specificity. Another potential limitation in our methodology was that LUS examinations were performed within 24 hours of chest radiographs. In critically ill children, respiratory status can evolve rapidly within this timeframe, potentially altering findings between the two imaging studies. This timing interval was implemented for logistical feasibility in the clinical setting. Nearly one-fourth of our study population consisted of post-operative cardiac patients, who may have altered pulmonary circulation that could influence LUS findings. To account for this, PARDS was defined and assessed according to the PALICC guidelines to minimize potential confounding.^(19)^ Another limitation is that our data set was heavily imbalanced, with only 21 negative cases (X-ray negative = 9.5%) out of 220 total. Of these, only four cases were correctly identified as negative by LUS, leading to very few true negatives (TNs). Additionally, a high proportion of children in our study presented with overlapping respiratory conditions, such as pneumonia with atelectasis or bronchiolitis with consolidation, which complicates interpretation and may have introduced classification bias.

This is one of the few studies describing LUS utility in children requiring varied levels of respiratory support in a pediatric ICU in a resource-limited setting. Our study findings are limited by being a single-center study with a small sample size, and some cases exhibited multiple overlapping pathophysiological processes. Current limitations include the lack of a standardized algorithm correlating LUS interpretation with clinical diagnoses, which can lead to inter-operator variability. A single clinician performed LUS interpretation, whereas a larger team could increase reliability and expertise. As part of our blinding methodology, LUS interpretation was performed without access to patient history or clinical progression, a scenario that differs from actual clinical practice and may have reduced the technique's diagnostic sensitivity. Notably, there were no cases of pneumothorax and a few with pleural effusion, conditions for which point-of-care LUS demonstrates diagnostic value. Additionally, some patients were not recruited due to personnel unavailability during study periods.

## CONCLUSION

In this single-center study of critically ill children, lung ultrasound demonstrated high sensitivity but low specificity compared with clinical diagnosis and chest X-rays. Lung ultrasound showed better diagnostic performance for pediatric acute respiratory distress syndrome; however, its poor agreement with chest X-rays and inconsistent specificity across diagnostic categories limit its use as a standalone diagnostic modality in the pediatric intensive care unit. Further studies to develop standardized protocols for image acquisition and interpretation, along with physician training, are necessary before its widespread use in resource-limited countries where chest X-rays or computed tomography may not be feasible.

## Data Availability

Data can be shared upon reasonable request to the corresponding author.
