# Supplementary material for: Diagnostic accuracy of lung ultrasound in children with respiratory pathology admitted in a pediatric intensive care unit of a low-resource setting: a single-center experience
Source: Crit Care Sci. 2026 Apr 16;38:e20260146. doi: 10.62675/2965-2774.20260146 (PMC13124121; doi:10.62675/2965-2774.20260146)
Supplement: Supplementary file 1 [file 2965-2774-ccsci-38-e20260146-suppl01.pdf]

Critical Care Science
https://doi.org/10.62675/2965-2774.20260146

SUPPLEMENTARY MATERIAL

Diagnostic accuracy of lung ultrasound in children with respiratory pathology admitted in a pediatric intensive care unit of a low-resource setting: a single-center experience

Qalab Abbas[1]*, Farah Khalid[1]*, Haania Rizwan[1], Uswah Siddiqi[2], Areesh Mevawalla[2], Arsheen Zeeshan[1], Fyezah Jehan[1]

Table 1S - Definitions of different terminologies and diagnoses

Clinical diagnoses	Definitions
Pneumonia	Acute infection of the pulmonary parenchyma that is associated with:<br>• 2 clinical findings (at least 2 of the following)<br>– Cough, new onset of respiratory secretions, dyspnea<br>– Auscultatory findings (rales, rhonchi, crackles, bronchial breathing, decreased breath sounds<br>– Hypoxemia<br>PLUS<br>• Vitals (fever 3 - 24 months ≥ 38.3ºC; > 2 years, > 38.0ºC; tachypnea [age defined])<br>AND<br>• Laboratory: WBC > 15,000/HPF and 10% > bands OR < 4,000/HPF<br>• Radiographic: infiltrate consistent with infection (interstitial, bronchial, alveolar), consolidation, cavitation, abscess, pneumatocele within 48 hours of admission
Bronchiolitis	A clinical syndrome of respiratory distress that occurs in children < 2 years of age and is characterized by upper respiratory symptoms (cough, rhinorrhea) leading to lower respiratory infection with inflammation, which results in wheezing and or crackles.<br>(Ref = UpToDate)
PARDS	Defined as per the Pediatric Acute Lung Injury Consensus Conference (PALICC 2015)*

Source: Pediatric Acute Lung Injury Consensus Conference Group. Pediatric acute respiratory distress syndrome: consensus recommendations from the Pediatric Acute Lung Injury Consensus Conference. Pediatr Crit Care Med. 2015;16(5):428-39.

This is an open access article under the CC BY license (https://creativecommons.org/licenses/by/4.0/).

Table 2S - Findings on lung ultrasound

Lung ultrasound findings	Definitions
Pleural line	It is a hyperechoic line below the rib-line
Lung sliding	Homogeneous twinkling (shimmering, sparkling to and for movement) of the pleural line with respiration. It represents the sliding of the visceral pleura against the parietal pleura
A-lines	These are hyperechoic horizontal lines that run parallel to the pleural lines. These are reverberations at a pleural-air interface, indicating the presence of air
B-lines	These are discrete laser-like, vertical hyperechoic lines originating from and perpendicular to the pleural lines, extending up to the bottom of the screen and moving synchronously with lung sliding
Confluent B-lines	These are non-discrete, laser-like, vertical hyperechoic lines (cannot be discreetly counted), with non-discrete origins from the pleural lines, extending to the bottom of the screen and moving synchronously with lung sliding
Z-lines	These are short, broad, ill-defined, vertical comet tail artifacts arising from the pleural line but not reaching the distal edge of the screen, and do not move with lung sliding
Interstitial syndrome	Multiple B-lines 7 mm apart, caused by thickened interlobular septa
Interstitial edema	Defined as when more than two B-lines are visible between two ribs
Alveolar edema	B-lines 3 mm or less apart are, by ground glass areas
Lung consolidation	A subpleural echo-poor (hypoechoic) circumscribed region, yielding the tissue-like sign (hepatization of the lung) and the shred sign (edge of the pleura looks irregular and shredded)<br>Small consolidation contained within one intercostal space and extending just below the pleural line (usually < 1cm)<br>Considerable consolidation extends to more than 1 intercostal space, extends beyond the pleural line, and may be associated with a pleural effusion. (usually < 1cm)
Interstitial abnormality	Sonologic findings:<br>Focal: Multiple B lines (> 3) present in a single view or unilaterally<br>Diffuse: Multiple B lines (> 3) present bilaterally
Atelectasis	Small areas of the collapsed part of the lung due to a decrease in the amount of air in the alveoli, resulting in volume loss and increased density<br>Sonologic findings:<br>Hypoechoic areas in the lung, triangular shape, associated with crowding of the bronchi due to loss of lung volume
Pleural effusion	Presence of fluid in the lateral pleural space between the lung and chest wall<br>Sonologic findings: anechoic space between visceral and parietal pleura<br>M-mode: presence of sinusoid sign

Table 3S - Criteria to diagnose different respiratory etiologies in pediatric chest X-rays

Lung pathology on chest X-ray	Findings identified on chest X-ray
Pediatric acute respiratory distress syndrome	Presence of bilateral infiltrates involving two or more quadrants on a frontal chest radiograph
Pneumonia	Presence of:<br>"End-point consolidation": a dense opacity that may be a fluffy consolidation of a portion or whole of a lobe or of the entire lung, often containing air bronchogram and sometimes associated with pleural effusion.<br>OR<br>"Other (non-endpoint) infiltrate": a linear and patchy density (interstitial infiltrate) in a lacy pattern involving both lungs, featuring peribronchial thickening and multiple areas of atelectasis, with lung inflation being normal to increased. It also includes minor patchy infiltrates that are not of sufficient magnitude to constitute primary end-point consolidation, and small areas of atelectasis, which in children can be challenging to distinguish from consolidation.
Bronchiolitis	Presence of hyperinflated lungs, hilar enlargement, perihilar or peribronchial infiltrates, diffuse interstitial infiltrates, subsegmental atelectasis, pulmonary infiltrates or primary atelectasis, cardiomegaly, pneumomediastinum, pneumothorax, and foreign body. The presence of any of these radiographic signs did not exclude the others.<br>We considered the first 5 signs compatible with "simple or uncomplicated AB". We considered the presence of the sixth sign compatible with "complicated AB".
Pulmonary edema	Presence of enlargement of the vascular pedicle width, defined as the superior mediastinum just above the aortic arch, and cephalization of pulmonary vessels
Atelectasis	Presence of linear increased density on chest x-ray. The apex tends to be at the hilum. The density is associated with volume loss. Some indirect signs of volume loss include vascular crowding or fissural, tracheal, or mediastinal shift, to PARDS the collapse. There may be compensatory hyperinflation of adjacent lobes, or hilar elevation (upper lobe collapse) or depression (lower lobe collapse). Segmental and subsegmental collapse may show linear, curvilinear, or wedge-shaped opacities.
Pleural effusion	Presence of fluid in the lateral pleural space between the lung and chest wall that is spatially associated with a pulmonary parenchymal infiltrate (including other infiltrate) or has obliterated enough of the hemithorax to obscure any infiltrate; in most cases, this will be seen at the costo-phrenic angle or as a layer of fluid adjacent to the lateral chest wall; this does not include fluid seen in the horizontal or oblique fissures.
Pneumothorax	A visible visceral pleural edge is a skinny, sharp, white line. No lung markings are seen peripheral to this line; the peripheral space is radiolucent compared to the adjacent lung. The lung may completely collapse. The mediastinum should not shift away from the pneumothorax unless a tension pneumothorax is present.
Other	Any other pathological finding besides the signs mentioned above.

Source: Schneider K. [Specific characteristics of chest Xray in childhood: basics for radiologists]. Radiologe. 2018;58(4):359-76. German.

Table 4S - Bayesian analysis of diagnostic accuracy parameters

Diagnostic metric	Posterior estimates	95% credible interval
Sensitivity, %	90.40	83.8% - 95.5
Specificity, %	21.80	7.9% - 40.5
ROC area (Sens. + Spec.)/2	0.57	0.46 - 0.67
Likelihood ratio (+) Pr(+|A)/Pr(+|N)	1.18	0.91 - 1.52
Likelihood ratio (-) Pr(-|A)/Pr(-|N)	0.38	0.11 - 1.27
Odds ratio LR(+)/LR(-)	3.09	0.79 - 12.22
Positive predictive value Pr(A|+),%	79.40	67.9% - 88.3
Negative predictive value Pr(N|-),%	44.40	13.7% - 78.8

Posterior estimates of sensitivity, specificity, likelihood ratios, and predictive values with 95% credible intervals from Bayesian analysis (Beta [1,1] priors).

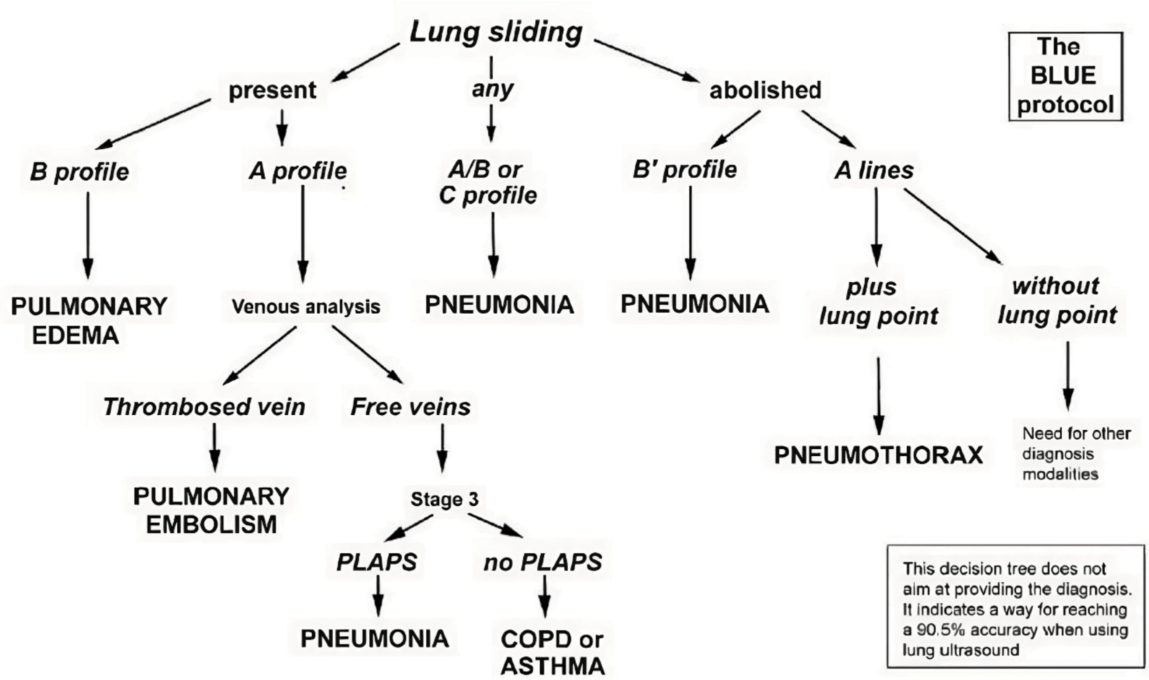

Figure 1S - Decision-making tree for lung ultrasound findings and different respiratory etiologies
Source: Lichtenstein DA, Mezière GA. Relevance of lung ultrasound in the diagnosis of acute respiratory failure. Chest. 2008;134(1):117-25.
